# Burden and Cost of Hospitalization for Respiratory Syncytial Virus in Young Children, Singapore

**DOI:** 10.3201/eid2607.190539

**Published:** 2020-07

**Authors:** Clarence C. Tam, Kee Thai Yeo, Nancy Tee, Raymond Lin, Tze Minn Mak, Koh Cheng Thoon, Mark Jit, Chee Fu Yung

**Affiliations:** National University Health System, Singapore (C.C. Tam, N. Tee);; National University of Singapore, Singapore (C.C. Tam, K.C. Thoon);; London School of Hygiene and Tropical Medicine, London, UK (C.C. Tam, M. Jit);; KK Women’s and Children’s Hospital, Singapore (K.T. Yeo, N. Tee, K.C. Thoon, C.F. Yung);; Duke-National University of Singapore Graduate Medical School, Singapore (K.T. Yeo, K.C. Thoon, C.F. Yung);; National Public Health Laboratory, Singapore (R. Lin, T.M. Mak);; Nanyang Technological University, Singapore (C.F. Yung)

**Keywords:** respiratory syncytial virus, respiratory infections, lower respiratory tract infection, pneumonia, bronchiolitis, disease burden, economic cost, economic modeling, viruses, Singapore

## Abstract

Respiratory syncytial virus (RSV) is the most common cause of pediatric acute lower respiratory tract infection worldwide. Detailed data on the health and economic burden of RSV disease are lacking from tropical settings with year-round RSV transmission. We developed a statistical and economic model to estimate the annual incidence and healthcare cost of medically attended RSV disease among young children in Singapore, using Monte Carlo simulation to account for uncertainty in model parameters. RSV accounted for 708 hospitalizations in children <6 months of age (33.5/1,000 child-years) and 1,096 in children 6–29 months of age (13.2/1,000 child-years). The cost of hospitalization was SGD 5.7 million (US $4.3 million) at 2014 prices; patients bore 60% of the cost. RSV-associated disease burden in tropical settings in Asia is high and comparable to other settings. Further work incorporating efficacy data from ongoing vaccine trials will help to determine the potential cost-effectiveness of different vaccination strategies.

Respiratory syncytial virus (RSV) is the commonest cause of acute lower respiratory tract infection in children <5 years of age worldwide, causing an estimated 33 million cases, 3 million hospitalizations, and 60,000 deaths annually (*1*). In young children and infants, RSV infections can cause bronchiolitis and pneumonia requiring hospitalization. In the United States, RSV accounts for 40% of respiratory hospitalizations in all age groups (*2*); children <5 years of age account for two thirds of this burden. Similarly, in the United Kingdom, RSV accounts for 28% of hospitalizations for lower respiratory tract infection in children <5 years of age (*3*).

Several RSV vaccines are in development. Potential immunization strategies include vaccination of children, pregnant mothers, and older adults. Assessing the potential impact and cost-effectiveness of future vaccination strategies relies on robust estimates of RSV-related health burden and cost, to inform national procurement and prioritization decisions. We present estimates of RSV-related primary care consultations, hospitalizations, and associated healthcare costs among children <30 months of age in Singapore.

## Methods

### Study Setting

Singapore, a high-income tropical country, has year-round RSV circulation. Compared with the distinct winter seasonal peaks in temperate settings, the RSV season is longer, peaking in May–September (*4*–*6*). Hospitalization costs in Singapore are borne by a mix of public and private insurance, out-of-pocket financing, and means-tested subsidies for lower-income patients. KK Women’s and Children’s Hospital (KKH) is the largest specialist women’s and children’s hospital in Singapore. All children admitted to KKH with respiratory symptoms, regardless of clinical severity, are tested by nasopharyngeal swab sampling for a panel of respiratory viruses using direct immunofluorescence antibody (DFA). Primary care is available through public polyclinics, private general practices, and pediatric clinics. Polyclinics are subsidized, and patients contribute a co-payment; consultations in private practices are paid out of pocket or through individual insurance. Approximately 80% of primary care consultations occur in the private sector (*7*).

### Approach

We used data on primary care consultations for acute respiratory illness (ARI) and hospital admissions for bronchiolitis and pneumonia in children <30 months of age, together with data on laboratory testing for RSV and healthcare bill sizes, to estimate incidence of medically attended RSV and associated healthcare costs from a societal perspective. We used Monte Carlo simulations to estimate numbers of primary care consultations and numbers of hospitalizations for bronchiolitis, pneumonia without complications, and pneumonia with complications. We developed 2 cost models: the full-cost model estimated the total cost of RSV-related healthcare, whereas the subsidized cost estimated the share of healthcare costs paid by patients after accounting for public subsidies.

### Data Sources

#### Bronchiolitis and Pneumonia Admissions

We obtained data on bronchiolitis and pneumonia hospitalizations in children <72 months of age during 2005–2014 from electronic inpatient admission records at KKH. We selected all admissions with diagnostic codes for pneumonia and bronchiolitis in any diagnostic field (International Classification of Diseases, 9th Revision [ICD-9], codes 480–486 and 466, or ICD, 10th Revision [ICD-10], codes J12–18 and J21). We excluded records for which a pathogen other than RSV was mentioned as the cause of bronchiolitis or pneumonia in the medical code description.

#### RSV and Influenza Positive Identifications

We extracted data on positive identifications of RSV and influenza at KKH from laboratory diagnostic records for children <30 months of age for the period 2005–2014. Viral infections were detected using DFA for influenza A and B viruses, RSV, adenovirus, parainfluenza viruses 1–3, and human metapneumovirus (D^3^ Double Duet DFA Respiratory Virus Screening and ID Kit; Diagnostic Hybrids; Quidel Corporation, https://www.quidel.com). The positive and negative percent agreements of virus detection by this test against a predicate test are 100%.

#### Rotavirus Positive Identifications

We used rotavirus as a negative control, to rule out spurious associations related to secular changes in recording of diagnoses or laboratory investigations. We extracted all positive identifications of rotavirus in children <30 months of age hospitalized for gastroenteritis during 2005–2014. Rotavirus detection in stool samples was done via an immunochromatographic assay (Simple Rotavirus/Stick Rotavirus; Operon, https://operon.es).

#### Public Primary Care Consultations

We obtained data on polyclinic consultations for ARI among children <5 years of age in 2014 from the Ministry of Health; the National Public Health Laboratory additionally tests for a range of respiratory pathogens in a subset of primary care ARI samples. We obtained data on the proportion of samples, by age group, in which RSV was detected as the sole pathogen as determined by a commercial multiplex assay (Seegene Allplex Respiratory Panel; Seegene Inc., http://www.seegene.com).

#### Costs of Hospitalization and Primary Care Consultation

Singapore Ministry of Health (MOH) standardized unit costs of hospitalization by condition, healthcare institution, and ward class, together with average length of stay (LOS), are published annually (*8*). Wards are categorized into 5 classes, A, B1, B2+, B2, and C, with A being unsubsidized and C being the most highly subsidized. We obtained published costs of an admission to KKH for bronchiolitis, pneumonia, and pneumonia with complications from the MOH website for the 5 different ward classes (Appendix Table 5). We determined the cost of a primary care consultation on the basis of the average cost of a polyclinic visit, which we obtained from the respective providers (*9*,*10*) (Appendix Table 4).

### Data Analysis

#### Proportion of Bronchiolitis and Pneumonia Admissions Attributed to RSV

We estimated the proportion of RSV-related bronchiolitis and pneumonia admissions using a seasonal regression method (*11*,*12*). We used a negative binomial model with an identity link to model weekly counts of bronchiolitis and pneumonia admissions against weekly counts of RSV-positive identifications for the years 2005–2013. We included an intercept term to account for admissions not explained by RSV. We fitted separate models for children <6 months of age and children 6–30 months of age. We used the model coefficients to predict the proportion of bronchiolitis and pneumonia admissions in 2014 attributable to RSV. We tested the model’s validity by comparing the model-predicted and observed values for 2014 and calculating the correlation coefficient (ρ), root mean squared error (RMSE), and mean absolute error (MAE) (Appendix Figure 1). In a sensitivity analysis, we ran additional regressions individually, adjusting for the following: linear and quadratic trend terms, weekly influenza positive identifications, weekly rotavirus positive identifications, and weekly gastrointestinal admissions (Appendix).

#### Percentage of Admissions for Pneumonia

Of admissions for bronchiolitis and pneumonia, the percentage specifically for pneumonia-related codes increased with age, which has implications for the cost of treatment; pneumonia incurs higher treatment costs. We used a logistic regression model with natural cubic splines of age and internal knots at 9-month age intervals to predict the age-specific proportion of pneumonia-related admissions in 2014.

#### LOS and Pneumonia with Complications

We modeled LOS in 2005–2013 by fitting an exponential regression to the percentage distribution of LOS separately for bronchiolitis and pneumonia admissions (Appendix Figure 4). We used the model coefficients to predict the LOS distribution for 2014.

We defined pneumonia with complications as pneumonia with LOS >5 days, assuming that pneumonia with complications results in longer hospitalization. Approximately 8% of admissions for bronchiolitis and pneumonia had a LOS >5 days, matching the percentage of admissions categorized as pneumonia with complications in MOH billing data.

#### Burden and Cost of RSV Hospitalization

We developed a model to estimate the annual number of hospitalizations for bronchiolitis, pneumonia, and pneumonia with complications and their associated cost, estimated separately for children <6 months and 6–29 months of age (Appendix). In the model, number of RSV-positive identifications in KKH in 2014 for each age group are inputs. We used the coefficients from the 3 regressions to estimate the overall number of bronchiolitis and pneumonia admissions attributable to RSV; the number of admissions that were bronchiolitis versus pneumonia by patient’s age in months; the number of admissions for pneumonia with complications, based on the predefined cutoff for LOS; and the total number of days of hospitalization for bronchiolitis, pneumonia, and pneumonia with complications. We extrapolated hospitalization estimates to the whole of Singapore by applying inflation factors to account for the proportion of hospitalizations occurring in other hospitals (Appendix, Equations 4.1–4.3).

To estimate the full hospitalization costs, we applied the average daily bill size in a class A (unsubsidized) ward to the total hospitalization days for bronchiolitis, pneumonia, and pneumonia with complications (Appendix Equation 5). To estimate the subsidized cost (the cost borne by patients), we applied the average daily bill size for each ward class to the number of hospitalization days spent by patients in each type of ward (Appendix Equation 6). We estimated costs separately for bronchiolitis, pneumonia, and pneumonia with complications.

#### Burden and Cost of RSV Primary Care Consultations

Because fine age stratification of primary care consultations was not available, we used the age distribution of RSV-positive identifications from KKH to infer the number of polyclinic consultations in children <6 months and 6–29 months of age (Appendix Equation 7). To estimate the number of consultations occurring in the private sector, we used the ratio of pediatric consultations (for children <5 years of age) in polyclinics versus private GPs, published in the 2014 Primary Care Survey (*7*). We estimated the proportion of respiratory consultations due to RSV using National Public Health Laboratory ARI testing data, in which 8% of ARI samples from children <5 years had RSV as the sole identified pathogen. We estimated the full cost of primary care consultations for RSV by applying the cost of an unsubsidized polyclinic consultation to the estimated number of consultations. To estimate the subsidized cost, we applied the cost of a pediatric polyclinic consultation to the subset of consultations occurring in government polyclinics (Appendix Equations 8.1–8.2).

#### Monte Carlo Simulation

To account for uncertainty in model parameters, we used Monte Carlo methods to sample parameter values at random from their assumed distributions. We performed 10,000 simulations. For each output parameter, we used the median and 2.5th and 97.5th percentiles of the sampled distributions as the point estimate and corresponding 95% CI.

To assess the influence of our definition of pneumonia with complications, we repeated the analyses varying the LOS cutoff used to define pneumonia with complications (+ 1 day). We adjusted costs to 2014 prices using the healthcare domain of the Consumer Price Index (*13*). We rounded cumulative healthcare costs to the nearest SGD 1,000.

We performed all analyses using Stata version 12 (Stata Corporation, https://www.stata.com) and R version 3.4.1 (*14*). The SingHealth Centralized Institutional Review Board approved this study (application 2017/2223).

## Results

**Figure 1:**
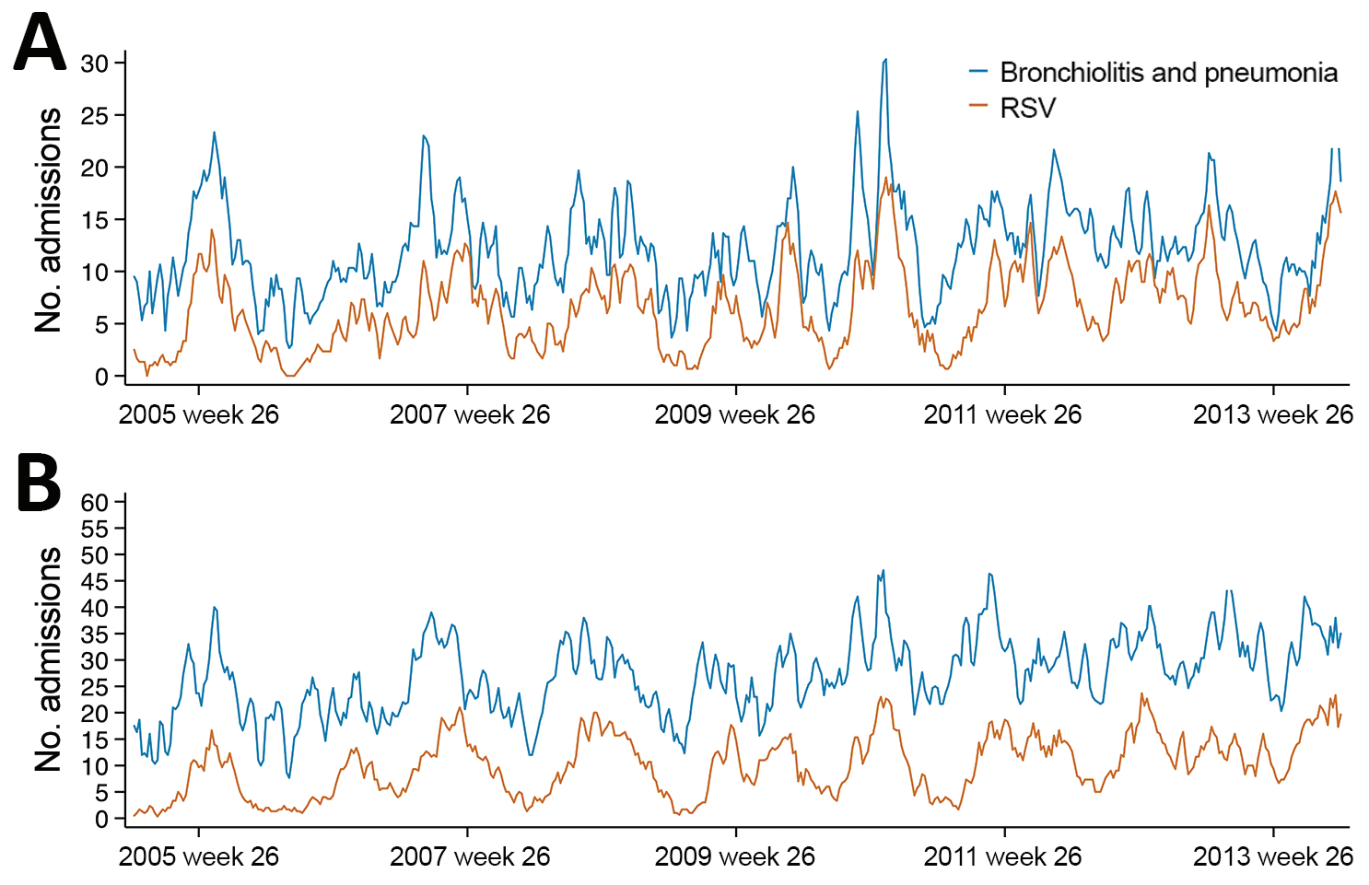
Hospital admissions for respiratory syncytial virus and for bronchiolitis and pneumonia over time by age group, Singapore, 2005–2013.

During 2005–2013, there were 18,323 bronchiolitis and pneumonia admissions in children <30 months of age at KKH, an average of 39 weekly admissions, and 7,691 RSV-positive identifications, a weekly average of 16. There was substantial temporal agreement between the two time-series, with a marked peak in RSV identifications in May–September in most years (Figure 1). Model validation showed high correlation between model-predicted and observed bronchiolitis admissions for 2014 (ρ = 0.88 for children <6 months of age; ρ = 0.81 for children 6–29 months of age) (Appendix Table 1).

### Hospitalizations Attributable to RSV

The negative binomial model with an intercept and a linear term for weekly RSV-positive identifications provided the best fit to the data. From this model, RSV accounted for 47.0% (95% CI 42.4%–51.5%) of bronchiolitis and pneumonia admissions among children <6 months of age, and 34.3% (95% CI 25.0%–33.1%) among children 6–29 months. The estimated yearly number of RSV-associated bronchiolitis admissions was 135–340 among children <6 months of age and 271–680 among children 6–29 months of age (Appendix Table 1).

Adjusting for linear or quadratic trend terms, influenza positive identifications, rotavirus positive identifications, and gastrointestinal admissions resulted in small changes to the RSV-attributable percentage. However, none of these more complex models provided a better fit to the data (Appendix Table 2).

### Percentage of Pneumonia Hospitalizations by Age

**Figure 2:**
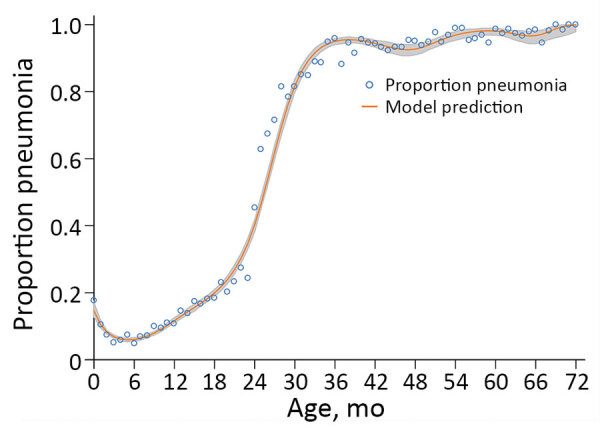
Proportion of bronchiolitis and pneumonia admissions for pneumonia-related codes as contrasted with model predictions by age, Singapore, 2005–2013. Gray shading along the curve indicates 95% CI.

Based on the regression with natural cubic splines, the percentage of pneumonia admissions was estimated to be 18.6% for children hospitalized in the first month of life. This percentage decreased to 5.5% by 4 months of age but rose to 74.0% by 29 months of age (Figure 2).

### Bronchiolitis and Pneumonia Hospitalization Rates

An estimated 708 (95% CI 664–765) bronchiolitis and pneumonia admissions due to RSV occurred in 2014 among children <6 months of age, a rate of 33.5 hospitalizations/1,000 child-years. For children 6–29 months, the corresponding number was 1,096 (95% CI 998–1,273), or 13.2 hospitalizations/1,000 child-years (Table 1). Among children <6 months of age, the number of admissions was 637 for bronchiolitis, 54 for pneumonia without complications, and 15 for pneumonia with complications. In children aged 6–29 months, the corresponding numbers were 826 for bronchiolitis, 203 for pneumonia without complications, and 63 for pneumonia with complications.

**Table 1:** Estimated RSV-associated hospitalizations and primary care consultations, Singapore, 2014

Age, mo	Outcome	Total no. cases (95% CI)	No. cases/1,000 person-years (95% CI)
Hospitalizations			
<6	All diagnoses	708 (664–765)	33.5 (31.4–36.2)
	Bronchiolitis	637 (604–671)	30.2 (28.6–31.8)
	Pneumonia	54 (30–99)	2.6 (1.4–4.7)
	Pneumonia with complications	15 (7–29)	0.7 (0.3–1.4)
6–29	All diagnoses	1,096 (994–1,269)	13.2 (12–15.3)
	Bronchiolitis	826 (793–862)	9.9 (9.5–10.4)
	Pneumonia	203 (115–372)	2.4 (1.4–4.5)
	Pneumonia with complications	63 (38–110)	0.8 (0.5–1.3)
Primary care consultations			
<6	ARI	3,600 (3,120–4,130)	170.5 (147.8–195.6)
6–29	ARI	5,700 (5,010–6,450)	68.6 (60.3–77.6)

*Estimates are expressed as the medians from 10,000 Monte Carlo simulations. Note that the sum of medians from individual diagnoses does not equal the median for all diagnoses combined. ARI, acute respiratory illness; RSV, respiratory syncytial virus.

### Primary Care Consultation Rates

The number of estimated primary care consultations for RSV among children <6 months of age was 3,600 (95% CI 3,120–4,130) or 170.5 consultations/1,000 child-years. The corresponding number of consultations in children 6–29 months was 5,700 (95% CI 5,010–6,450), for a rate of 68.6 consultations/1,000 child-years (Table 1).

### Hospitalization Costs

**Figure 3:**
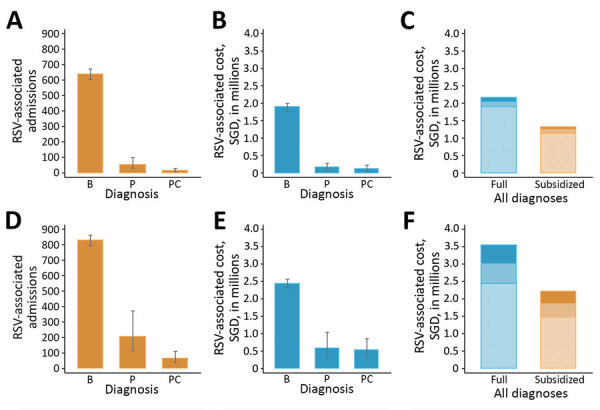
Estimated annual RSV-associated hospital admissions and costs for children <5 months of age (A–C) and children 6–29 months (D–F), Singapore, 2005–2013. Panels show estimated annual RSV-associated hospital admissions (panels A, D), total hospitalization costs by diagnosis (B, E), and full vs. subsidized costs (C, F). For panels C and F, shading indicates, from lightest to darkest: bronchiolitis, pneumonia without complications, pneumonia with complications. Point estimates and error bars representing medians and central 95% CI distributions were generated from 10,000 Monte Carlo simulations. B, bronchiolitis; P, pneumonia; PC, pneumonia with complications; RSV, respiratory syncytial virus; SGD, Singapore dollars.

The annual unsubsidized cost of RSV-associated bronchiolitis and pneumonia hospitalizations among children <30 months of age was SGD 5.7 million (95% CI SGD 5.2 – SGD 6.4 million). Patients bore SGD 3.6 million (US $2.6 million), or 63%, of the total cost (Table 2). Approximately 40% of admissions occurred in maximally subsidized class C wards and 30% in unsubsidized class A wards (Figure 3).

**Table 2:** Cost of RSV-associated hospitalizations and primary care consultations, Singapore, 2014*

Age, mo	Outcome	Full cost (95% CI)	Subsidized cost (95% CI)
Hospitalizations			
<6	All	$2,160,000 ($2,002,000–$2,352,000)	$1,321,000 ($1,168,000–$1,492,000)
	Bronchiolitis	$1,881,000 ($1,771,000–$1,995,000)	$1,127,000 ($1,006,000–$1,250,000)
	Pneumonia	$152,000 ($82,000–$278,000)	$106,000 ($53,000–$198,000)
	Pneumonia with complications	$119,000 ($55,000–$220,000)	$80,000 ($25,000–$167,000)
6–29	All	$3,554,000 ($3,175,000–$4,118,000)	$2,236,000 ($1,932,000–$2,651,000)
	Bronchiolitis	$2,436,000 ($2,319,000–$2,563,000)	$1,459,000 ($1,328,000–$1,600,000)
	Pneumonia	$573,000 ($321,000–$1,041,000)	$401,000 ($217,000–$729,000)
	Pneumonia with complications	$523,000 ($322,000–$857,000)	$358,000 ($191,000–$610,000)
Primary care consultations			
<6	Primary care attendances	$177,000 ($153,000–$203,000)	$118,000 ($102,000–$136,000)
6–29	Primary care attendances	$280,000 ($246,000–$317,000)	$187,000 ($163,000–$213,000)
Hospitalizations and primary care consultations			
<6	All	$2,337,000 ($2,175,000–$2,530,000)	$1,440,000 ($1,285,000–$1,611,000)
6–29	All	$3,833,000 ($3,454,000–$4,399,000)	$2,423,000 ($2,115,000–$2,838,000)
<30	All	$6,228,000 ($5,734,000–$6,950,000)	$3,899,000 ($3,506,000–$4,432,000)

*Estimates are expressed as the medians from 10,000 Monte Carlo simulations. Note that the sum of medians from individual diagnoses does not equal the median for all diagnoses combined. All costs are in Singapore dollars. RSV, respiratory syncytial virus.

Among children <6 months of age, average cost per bronchiolitis hospitalization was SGD 2,953 (US $2,209), rising to SGD 7,944 (US $5,942) for a hospitalization for pneumonia with complications. Among children 6–29 months of age, average costs were SGD 2,949 (US $2,206) for bronchiolitis and SGD 8,300 (US $6,208) for pneumonia with complications. Varying the definition of pneumonia with complications by +1 day from the 5-day cutoff had negligible effect on estimates of case counts or overall cost (Appendix Figure 5).

### Primary Care Costs and Costs per Child

The annual cost of primary care consultations was SGD 0.46 million (US $0.34 million), of which 38% was incurred for children <6 months of age. The mean cost per case was SGD 49 (US $37).

The overall cost of RSV-related hospitalizations and primary care consultations was SGD 6.2 million (US $4.7 million). This total is equivalent to SGD 60 annually per birth (US $45), of which SGD 55 represents hospitalization costs.

## Discussion

RSV causes substantial pediatric health and economic burden in Singapore, accounting for 33.5 hospitalizations/1,000 child-years among children <6 months of age and 13.2 hospitalizations/1,000 child-years in children 6–29 months of age. The annual healthcare cost attributable to RSV is SGD 6.2 million (US $4.7 million), or SGD 60 (US $45) per birth, the bulk of which is for acute hospital care. Our findings help to address the gap in information to support the cost-effectiveness evaluations of future RSV vaccination strategies in tropical settings.

Shi et al. reported similar estimates on community incidence of RSV disease (66/1,000 children) and hospitalization (26/1,000 children) for infants <6 months in high-income countries (*1*). RSV hospitalization rates in children <6 months from 4 East Asia and Asia-Pacific studies ranged from 14 hospitalizations/1,000 children/year in 2 rural provinces in Thailand to 42.7/1,000/year in Alice Springs, Northern Territory, Australia (*1*). Homaira et al., using linked administrative health data from Australia, estimated a hospitalization rate of 26/1,000 children <3 months (*15*). RSV hospitalization rates of ≈45/1,000 children <6 months of age have been reported in England and Wales (*11*) and Denmark (*16*). Our estimate is somewhat lower, at 33/1,000 children. This difference could reflect differences in estimation methods or the lower fertility rates in Singapore, which might result in reduced transmission of RSV among very young children.

Hospitalization costs are difficult to compare because of differences between countries in healthcare financing. In our analysis, the hospitalization cost per child was ≈US $41, higher than in Denmark (≈US $25) (*16*) but lower than in England and Wales (≈US $82) (*11*). In our analysis, two thirds of hospitalization costs were borne by patients through personal insurance, Medisave (a national medical savings scheme), or out-of-pocket payments. In health systems with shared financing, determining the share of the cost borne by different sectors can be relevant for policy decisions. Although the healthcare costs of disease are shared by patients, insurers, and governments, it is a country’s government that is generally responsible for decisions about vaccine introduction and financing. Clarifying how the costs of averted illness affect different parties can help to inform vaccine policy decisions.

A limitation of our analysis is a lack of openly available information on how closely hospital bill amounts reflect actual treatment costs for RSV disease. We used average bill sizes for services in public-sector hospitals and polyclinics, which are likely to reflect treatment costs more closely than do bills for services from private healthcare providers, which are likely to include additional profit margins. Despite the large burden and cost we identified, our figures are likely to be underestimates. First, we limited our analysis to children <30 months of age because we lacked data on positive RSV identifications in older children. Second, our primary care estimates exclude consultations to private pediatricians, which were not included in the Primary Care Survey. Third, although we obtained detailed estimates of RSV hospitalization costs by ward class, our estimates of bill sizes in private hospitals is conservative. Because hospital billing data were not available by age group, we estimated the proportion of pediatric RSV admissions in private hospitals using the distribution of bronchiolitis admissions treated in different ward types, because bronchiolitis is primarily a pediatric diagnosis. We could not apply hospital-specific bill sizes because the cost of pneumonia admissions is usually heavily influenced by adult admissions; therefore, we applied KKH bill sizes for (unsubsidized) class A wards to all ward types as a proxy for the actual cost to providers of hospital care. Finally, we did not consider societal costs resulting from time taken off work by caretakers or nonhealthcare expenditure resulting from illness.

We did not estimate the fraction of RSV burden that occurs in higher-risk groups, such as preterm babies or children with underlying conditions, which could have implications for subsequent assessment of vaccination strategies. However, the overall healthcare cost is unlikely to be affected. Although we might have overestimated the burden attributable to RSV because of possible concurrent infections with other pathogens causally related to respiratory illness, we believe this is unlikely. We estimated RSV impact on primary care using the proportion of ARI samples in which RSV was identified as the sole pathogen (≈75% of samples in which RSV was detected). In estimating hospitalization burden, we used a regression approach that accounts for the seasonal correlation between hospital admissions and RSV-positive tests. To substantially affect our estimates, much of this seasonal variability would need to result from infections with other pathogens sharing similar seasonal patterns, but that is highly unlikely. Influenza showed very little correlation with bronchiolitis and pneumonia hospitalization data, whereas pneumococcal disease, also important in this age group, accounts for only a modest number of hospitalizations in Singapore (≈150 annually).

Strengths of our analysis include the availability of a long time-series of RSV-positive identifications based on routine, systematic diagnostic testing of pediatric respiratory admissions, and on patient-level data for diagnosis and length of hospitalization. The average hospitalization cost was US $2,200 for bronchiolitis and $6,000 for pneumonia. Studies of RSV-associated healthcare costs in similar Asia settings are lacking; Sruamsiri et al. estimated the average cost of RSV-associated hospitalization in Japan at US $3,300 (*17*) and Homaira et al. in Australia at US $4,500 (*15*). Of note, we estimated the cost borne by patients and the health sector; in hybrid public–private healthcare financing systems, future vaccination policy options may be better informed by understanding how avoidable costs affect different sectors of society. Our results indicate that patients bear >60% of hospitalization costs either through health insurance schemes or out-of-pocket payments.

Our findings add to the increasing body of data on the burden of RSV in infants and young children in both high- and low-income settings, and point to the need and potential for RSV vaccines to reduce neonatal disease burden. As evidence for the efficacy of new RSV vaccines emerges from ongoing trials, these data will provide a much-needed baseline against which to measure the cost effectiveness of different vaccination strategies.

Appendix Additional information about respiratory syncytial virus in young children, Singapore.
