# Supplementary material for: Burden and Cost of Hospitalization for Respiratory Syncytial Virus in Young Children, Singapore
Source: Emerg Infect Dis. 2020 Jul;26(7):1489–96. doi: 10.3201/eid2607.190539 (PMC7323550; doi:10.3201/eid2607.190539)
Supplement: Appendix — Additional information about respiratory syncytial virus in young children, Singapore. [file 19-0539-Techapp-s1.pdf]

Article DOI: https://doi.org/10.3201/eid2607.190539

Burden and Cost of Hospitalization for Respiratory Syncytial Virus in Young Children, Singapore

Appendix

In this document, we describe the statistical methods used to estimate the burden and cost of RSV hospitalization. To simplify the notation, we limit the text to estimation methods for children <6 months of age, but the same methods were used for the older age group of 6–29 months.

Estimating RSV-attributable Hospitalizations Using Seasonal Regression

We estimated the number and proportion of hospitalizations for bronchiolitis and pneumonia attributable to RSV by regressing the weekly number of hospital admissions against the weekly number of RSV-positive identifications for the years 2005–2013 (Appendix Figure 2), using a regression model of the form:

$H_{KKH,w} = a + \mu_r R_w$ (Equation 1)

where H_w is the weekly number of hospitalizations for bronchiolitis and pneumonia at KK Women's and Children's Hospital (KKH) and R_w represents the number of RSV-positive identifications. The coefficient, μ_r, measures the change in weekly bronchiolitis and pneumonia hospitalizations per unit change in RSV-positive identifications; the intercept, a, captures the hospitalization burden attributable to pathogens other than RSV. The regression was fitted using a negative binomial model with identity link, as previously used by Cromer et al. (*1*). The estimated coefficient and associated standard error were used to estimate the RSV-attributable hospitalization burden for 2014 in subsequent simulations.

In a series of sensitivity analyses, we accounted for long-term secular trends by including both linear and quadratic terms for week in the regression model. In addition, to account for possible changes to the epidemiology of RSV during the 2009 influenza pandemic, we fitted additional models excluding data for 2009, both without a trend term, and with separate trend

terms for the years 2005–2008 and 2010–2013. We also modeled the effect of influenza by including weekly influenza positive identifications in the model. Finally, we fitted models including weekly rotavirus positive identifications and weekly gastrointestinal admissions as negative controls. For each of these models, we assessed model fit using Bayesian information criterion (BIC), favoring models with a lower BIC value.

Estimating the proportion of hospitalizations due to pneumonia by age

Preliminary analysis of hospitalization data indicated that, although the overall number of bronchiolitis and pneumonia hospitalizations decreases markedly with age, the proportion of these due to pneumonia (as opposed to bronchiolitis) increased with age (Appendix Table 3). Accounting for this is important to avoid underestimating RSV burden and cost in older age groups. We modeled age-dependent changes in the proportion of pneumonia hospitalizations between 2005–2013 with a logistic regression model:

$$\alpha_m = ln\left(\frac{p_m}{1-p_m}\right) = b_0 + \sum_{k=1}^{k=9} b_k S_k \text{ (Equation 2)}$$

where the outcome, α_m, is the log odds of a pneumonia hospitalization at age *m* months and the explanatory variables, S_k, are regressors corresponding to natural cubic splines of age (over the range 0–72 months of age) with $k = 9$ equally spaced knots. The number of knots was decided based on a visual inspection of plots of observed and model-predicted values, so as to retain the minimum number of knots possible while capturing salient features in the observed data. We used model-predicted values and associated uncertainty to estimate the age-specific proportion of pneumonia hospitalizations for 2014 in subsequent simulations.

Modeling length of hospital stay (LOS)

The distribution of LOS was heavily right-skewed with the mode at 2 days. Because pneumonia hospitalizations tend to be longer, we modeled LOS separately for bronchiolitis and pneumonia hospitalizations during 2005 and 2013, using an exponential regression with 2 terms to allow for the smaller proportion of hospitalization with LOS of 1 day relative to 2 days:

$$ln(l_{o,d}) = b_{0,o} + b_{1,o}L_1 + b_{2,o}L_2 \text{ (Equation 3)}$$

where $l_{o,d}$ represents the proportion of hospitalizations for outcome *o* (bronchiolitis or pneumonia) that had LOS equal to *d* days. The terms L_1 and L_2 are defined as:

$$L_1 = \begin{cases} 1, d = 1 \\ 2, d \geq 2 \end{cases}$$

$$L_2 = \begin{cases} 0, d < 3 \\ d - 2, d \geq 3 \end{cases}$$

Estimating RSV Hospitalization Burden and Cost in 2014

RSV-Attributable Hospitalizations

We estimated age-specific hospitalizations for bronchiolitis and pneumonia attributable to RSV in 2014 by multiplying the number of RSV-positive identifications by a factor *r* derived from Equation 1 above:

$$H_{KKH,RSV,m} = R_m \times r$$

$$r \sim N(\mu_r, \sigma_r)$$

Hospitalizations for Bronchiolitis and Pneumonia

We estimated the age-specific number of pneumonia hospitalizations by multiplying the overall number of hospitalizations at age *m* months, $H_{RSV,m}$, by the the age-specific proportion of these hospitalizations that are due to pneumonia, using parameters derived from Equation 2. The number of bronchiolitis hospitalizations was derived by subtraction:

$$P_{KKH,m} = H_{KKH,RSV,m} \times p_m$$

$$B_{KKH,m} = H_{KKH,RSV,m} - P_{KKH,m}$$

$$p_m = \frac{e^{\alpha_m}}{e^{\alpha_m} + 1}$$

$$\alpha_m \sim N\left(\mu_{\alpha_m}, \sigma_{\alpha_m}\right)$$

The total pneumonia and bronchiolitis hospitalizations was obtained by summing over age categories:

$$P_{KKH} = \sum_{m=0}^{m=5} P_{KKH,m}$$

$$B_{KKH} = \sum_{m=0}^{m=5} B_{KKH,m}$$

We then estimated the distribution of bronchiolitis and pneumonia cases by length of stay:

$$P_{KKH,d} = P_{KKH} \times l_{o=p,d}$$
$$B_{KKH,d} = B_{KKH} \times l_{o=b,d}$$
$$ln(l_{o,d}) = b_{0,o} + b_{1,o}L_1 + b_{2,o}L_2$$
$$b_0 \sim N(\mu_{b_0}, \sigma_{b_0})$$
$$b_1 \sim N(\mu_{b_1}, \sigma_{b_1})$$
$$b_2 \sim N(\mu_{b_2}, \sigma_{b_2})$$

where $P_{KKH,RSV,d}$ and $B_{KKH,RSV,d}$ represent the number of RSV-related pneumonia hospitalizations with length of stay equal to *d* days and $l_{o\,=\,p,d}$ and $l_{o\,=\,b,d}$ are the corresponding proportions of hospitalizations with length of stay equal to *d* days, derived from Equation 3. In the base case scenario, we defined pneumonia with complications as pneumonia hospitalizations with LOS greater than 5 days:

$$PN_{KKH} = \sum_{d=1}^{d=5} P_{KKH,d}$$
$$PC_{KKH} = \sum_{d=6}^{d=366} P_{KKH,d}$$

such that *PN* and *PC* represent the number of pneumonia hospitalizations with and without complications respectively.

Next, we estimated the number of admissions occurring at KKH by ward class:

$$\boldsymbol{B}_{KKH,w} \sim Mult(\boldsymbol{w}_B, B_{KKH})$$

where $\boldsymbol{B}_{KKH,w}$ is a vector representing the number of bronchiolitis admissions in each of 5 ward classes at KKH. $\boldsymbol{B}_{KKH,w}$ is drawn from a multinomial distribution with parameters $\boldsymbol{w}_B$, a vector representing the proportions of bronchiolitis admissions occurring in each ward class (Appendix Table 5), and B_{KKH}, the number of RSV-related bronchiolitis admissions as defined above. The corresponding ward-specific admissions for uncomplicated pneumonia and pneumonia with complications were obtained analogously using corresponding multinomial distributions, $\boldsymbol{w}_{PN}$ and $\boldsymbol{w}_{PC}$.

To account for pediatric admissions occurring in hospitals other than KKH, we multiplied our estimates by a set of inflation factors. These factors were derived from the proportion of bronchiolitis cases in Singapore admitted to KKH, as reported in Ministry of Health (MOH) hospital billing data (*2*). We used bronchiolitis admissions to derive these inflation factors because billing data are not available by age group. Only a subset of public and private tertiary hospitals have pediatric departments, and bronchiolitis is likely to be almost exclusively a pediatric diagnosis. The estimated inflation factors are thus likely to reflect more accurately the ratio of pediatric admissions seen at KKH relative to other hospitals, whereas pneumonia admissions will include a large fraction of adult patients. The inflation factors were ward class–specific. This is important because the distribution of ward classes varies between hospitals; subsidized wards are only available in public-sector hospitals.

$B_w = \sum_{w=1}^{w=5} B_{KKH,w} * I_w$ (Equation 4.1)

$PN_w = \sum_{w=1}^{w=5} PN_{KKH,w} * I_w$ (Equation 4.2)

$PC_w = \sum_{w=1}^{w=5} PC_{KKH,w} * I_w$ (Equation 4.3)

Here, B_w is the number of bronchiolitis admissions in ward class w across all Singapore hospitals. I_w is a ward class-specific inflation factor. Estimates for pneumonia with and without complications are derived analogously.

To estimate the hospitalization costs, we first multiplied the number of admissions (for each diagnosis and ward class) by the average duration of hospitalization:

$D_{B,w} = B_w * d_B$

$D_{B,w}$, the total bronchiolitis hospitalization days among patients in ward class w, is obtained by multiplying the total number of bronchiolitis admissions in wards of class w by the average duration of hospitalization for bronchiolitis patients. The total days of hospitalization in each ward class for uncomplicated and complicated pneumonia admissions were derived analogously. The full (unsubsidised) cost of hospitalization was then:

$T_B = \sum_{w=1}^{w=5} D_{B,w} * s_{B,w=1}$ (Equation 5)

where T_B represents the total cost of bronchiolitis hospitalizations across all ward classes and $s_{B,w=1}$ is the average cost per day of hospitalization for a bronchiolitis patient admitted in

class A (private wards), obtained from MOH billing data for admissions to KKH. Ward-specific costs for uncomplicated and complicated pneumonia admissions were similarly derived.

The subsidized cost (that borne by patients through insurance or out-of-pocket payments) was estimated by applying the ward class–specific mean daily hospitalization costs, $s_{B,w}$:

$S_B = \sum_{w=1}^{w=5} D_{B,w} * s_{B,w}$ (Equation 6)

Costs for uncomplicated and complicated pneumonia were derived analogously.

Estimating Primary Care Consultations Due to RSV

We obtained data on the number of acute respiratory infection (ARI) consultations to government polyclinics among children <5 years of age from the Ministry of Health. We also obtained data from the National Public Health Laboratory on RSV testing results from microbiological surveillance of primary care ARI consultations. Samples from ARI patients are submitted for microbiological testing by participating sentinel primary care centers. These samples have been tested using a commercial multiplex PCR assay (Seegene Allplex Respiratory Panel Assays; http://seegene.com). Between 2012–2017, 3,402 samples were tested from children <5 years, of which 8.2% were positive for RSV with no other pathogen identified. To estimate the number of polyclinic consultations attributable to RSV, we applied the proportion of samples positive for RSV to the number of polyclinic consultations as follows:

$$F = \frac{K}{M} * G$$

$$K \sim Binom(\pi_1, M)$$

where π_1 is the proportion of samples testing positive for RSV, M is the total number of tests performed on samples from children aged <6 months, and G is the number of ARI consultations in polyclinics.

To account for primary care consultations in the private sector, we estimated the ratio of pediatric consultations in polyclinics versus private GPs, using data on the proportion of consultations among children <5 years in polyclinics and private GPs as reported by MOH in the 2014 Primary Care Survey (*3*). We assumed binomial distributions for these proportions:

$$q = \frac{Q}{G} + 1$$

$$R = q * G$$

$$Q \sim Binom(\pi_2, W)$$

Here, q is a multiplier that inflates the number of polyclinic consultations, G, to account for private sector consultations. Q represents the number of private GP consultations by children aged <6 months among GP clinics participating in the 2014 Primary Care Survey. Q is assumed to have a binomial distribution dependent on W, the daily number of private GP consultations, and π_2, the proportion of these occurring among children <5 years. R is an estimate of the total number of primary care consultations for RSV, both among polyclinics and private GP clinics.

Because primary care ARI consultation data were not available in fine age strata, we estimated the age distribution of primary consultations among children <5 years by month of age. We assumed that this age distribution was similar to the age distribution of RSV-positive identifications at KKH. We fitted a logarithmic function to the age-related RSV data (Appendix Figure 3) and applied this function to the estimated number of RSV-related primary care consultations to estimate the proportion of primary care consultations by month of age:

$$ln(R_m) = i_0 + i_1 R_1 + i_2 R_2 + i_3 R_3 \text{ (Equation 7)}$$

where R_m represents the number of RSV-related polyclinic consultations among children aged m months, R_1 to R_3 are age group indicators, i_1 to i_3 the corresponding regression coefficients and i_0 is a constant term. The terms R_1 to R_3 are defined as:

$$R_1 = \begin{cases} 0, m = 0 \\ 1, m \geq 1 \end{cases}$$

$$R_2 = \begin{cases} 0, m < 2 \\ 1, m = 2 \\ 2, m > 2 \end{cases}$$

$$R_3 = \begin{cases} 0, m < 3 \\ m - 3, m \geq 3 \end{cases}$$

The number of RSV-related primary care consultations among children aged <6 months is:

$$C = c * R$$

$$c = \frac{\sum_{m=0}^{m=5} R_m}{R}$$

with c representing the proportion of RSV-related consultations that occur in children aged <6 months.

We estimated the full cost of consultations by applying the unsubsidized cost of a polyclinic consultation to the estimated number of primary care visits due to RSV. To estimate the subsidized cost, we applied the cost of a pediatric consultation:

$U = C * u$ (Equation 8.1)

$V = C * v$ (Equation 8.2)

where U and V represent respectively the full and subsidized cost of all RSV-related primary care consultations in children <6 months of age, *u* is the full cost of a polyclinic consultation, and *v* is the subsidized cost of a pediatric polyclinic consultation.

References

<jrn>1. Cromer D, van Hoek AJ, Newall AT, Pollard AJ, Jit M. Burden of paediatric respiratory syncytial virus disease and potential effect of different immunisation strategies: a modelling and cost-effectiveness analysis for England. Lancet Public Health. 2017;2:e367–74. https://doi.org/10.1016/S2468-2667(17)30103-2</jrn>

<eref>2. Singapore Ministry of Health. Fee benchmarks and bill amount information. [cited 2018 Dec 4]. https://www.moh.gov.sg/cost-financing/fee-benchmarks-and-bill-amount-information</eref>

<eref>3. Singapore Ministry of Health. Primary care survey 2014. 2017 [cited 2019 Jan 16]. https://www.moh.gov.sg/resources-statistics/reports/primary-care-survey-2014-report</eref>

Appendix Table 1. Distribution of RSV-positive identifications at KK Women's and Children's Hospital, Singapore, by sex, age and year

Variable	No. RSV-positive identifications (N = 18,428)	% RSV positive
Sex		
M	11,488	62.3
F	6,940	37.7
Age, mo		
<1	449	2.4
1	1,154	6.3
2	1,197	6.5
3	932	5.1
4	999	5.4
5	985	5.3
6	1,077	5.8
7	1,026	5.6
8	971	5.3
9	875	4.7
10	786	4.3
11	742	4.0
12	757	4.1
13	620	3.4
14	632	3.4
15	556	3.0
16	518	2.8
17	450	2.4
18	462	2.5
19	460	2.5
20	468	2.5
21	480	2.6
22	420	2.3
23	382	2.1
24	250	1.4
25	179	1.0
26	166	0.9
27	146	0.8
28	138	0.7
29	151	0.8
Year		
2005	1,818	9.9
2006	1,480	8.0
2007	2,001	10.9
2008	2,075	11.3
2009	1,807	9.8
2010	2,236	12.1
2011	2,362	12.8
2012	2,251	12.2
2013	2,398	13.0

Appendix Table 2. Estimated bronchiolitis and pneumonia admissions in KK Women's and Children's Hospital, Singapore, by year and age group

Year	<6 mo (95% CI)	6–29 mo (95% CI)
2005	231 (209–252)	271 (209–252)
2006	149 (135–162)	257 (135–162)
2007	311 (283–340)	495 (283–340)
2008	291 (264–318)	553 (264–318)
2009	247 (225–270)	421 (225–270)
2010	334 (303–365)	501 (303–365)
2011	363 (330–397)	569 (330–397)
2012	368 (334–403)	585 (334–403)
2013	375 (340–409)	680 (340–409)

Appendix Table 3. Observed and model-predicted percentage of all bronchiolitis and pneumonia admissions due to pneumonia by age, KK Women's and Children's Hospital, Singapore, 2005–2013*

Age, mo	Observed %	Predicted % (95% CI)*
0	18.1	18.6 (15.7–21.9)
1	11.2	10.8 (9.7–12.0)
2	7.0	7.0 (6.1–8.0)
3	4.8	5.7 (5.0–6.4)
4	6.1	5.5 (4.8–6.4)
5	7.1	6.0 (5.1–7.0)
6	5.1	6.6 (5.8–7.4)
7	7.4	7.2 (6.3–8.3)
8	7.0	7.7 (6.8–8.8)
9	10.6	8.3 (7.4–9.3)
10	9.7	9.2 (8.1–10.5)
11	10.6	10.8 (9.7–12.1)
12	10.3	12.8 (11.7–14.0)
13	13.9	14.4 (13.1–15.9)
14	13.4	15.1 (13.6–16.7)
15	17.3	15.0 (13.7–16.4)
16	17.4	14.8 (13.7–16.0)
17	18.0	15.1 (13.9–16.3)
18	17.3	16.4 (15.1–17.8%)
19	23.9	18.9 (17.5–20.5)
20	22.2	22.7 (21.1–24.3)
21	24.4	27.6 (26.1–29.2)
22	28.6	33.7 (32.0–35.3)
23	25.7	40.3 (38.5–42.2)
24	45.2	47.1 (45.0–49.2)
25	61.2	53.6 (51.3–55.9)
26	66.3	59.6 (57.2–62.0)
27	69.9	65.1 (62.7–67.3)
28	79.3	69.9 (67.6–72.0)
29	74.5	74.0 (72.0–76.0)
30	81.7	77.6 (75.7–79.4)
31	84.2	80.6 (78.9–82.2)
32	83.6	83.2 (81.6–84.6)
33	88.1	85.3 (83.8–86.7)
34	87.1	87.1 (85.7–88.4)
35	94.1	88.6 (87.3–89.9)
36	95.1	89.9 (88.6–91.1)
37	87.4	91.0 (89.7–92.1)
38	93.6	91.9 (90.6–93.0)
39	89.5	92.7 (91.4–93.7)
40	96.0	93.3 (92.1–94.4)
41	94.9	93.9 (92.8–94.9)
42	93.8	94.4 (93.3–95.3)
43	92.1	94.8 (93.8–95.7)
44	91.2	95.2 (94.2–96.0)
45	92.5	95.5 (94.5–96.3)
46	92.5	95.7 (94.8–96.5)
47	94.7	96.0 (95.1–96.7)
48	94.3	96.2 (95.4–96.9)
49	92.9	96.3 (95.6–97.0)
50	94.2	96.5 (95.7–97.1)

Age, mo	Observed %	Predicted % (95% CI)*
51	98.7	96.6 (95.9–97.2)
52	94.4	96.7 (96.0–97.3)
53	96.6	96.8 (96.1–97.4)
54	98.9	96.9 (96.2–97.5)
55	98.9	96.9 (96.2–97.5)
56	94.9	97.0 (96.3–97.6)
57	96.2	97.0 (96.3–97.6)
58	98.6	97.1 (96.3–97.7)
59	93.2	97.1 (96.3–97.7)
60	98.4	97.1 (96.2–97.8)
61	97.0	97.1 (96.2–97.8)
62	98.6	97.1 (96.1–97.9)
63	97.1	97.1 (96.0–97.9)
64	96.3	97.1 (95.9–97.9)
65	97.3	97.1 (95.8–98.0)
66	98.2	97.0 (95.6–98.0)
67	93.2	97.0 (95.5–98.0)
68	97.7	97.0 (95.3–98.1)
69	100.0	96.9 (95.1–98.1)
70	98.0	96.9 (95.0–98.1)
71	100.0	96.9 (94.8–98.2)
72	100.0	96.8 (94.5–98.2)

*Percentage estimated from the regression model in Equation 2 (see also Figure 2)

Appendix Table 4. Sensitivity analysis for RSV-attributable burden by negative binomial regressions for 2005–13 data

Age group, mo	Model*	Description	β†	se(β)	BIC‡	% RSV§	No. RSV admissions¶
<6 mo	1	No trend	0.8907	0.0439	−2348.104	47.0%	5686
	2	Linear trend	0.9018	0.0461	−2341.217	47.6%	5686
	3	Quadratic trend	0.9041	0.0462	−2335.750	47.7%	5686
	4	Exclude 2009 and no trend	0.9137	0.0464	−2039.162	47.9%	5716
	5	Exclude 2009 and two trend terms	0.9174	0.0488	−2026.919	48.0%	5730
	6	Include rotavirus	0.9050	0.0444	−2343.507	47.7%	5686
	7	Include GI admissions	0.8893	0.0439	−2342.419	46.9%	5686
	8	Include influenza	0.8710	0.0448	−2346.221	45.9%	5687
6–29 mo	1	No trend	0.9238	0.0543	−2322.363	34.3%	12647
	2	Linear trend	0.7832	0.0562	−2316.329	29.1%	12646
	3	Quadratic trend	0.7834	0.0561	−2308.695	29.1%	12645
	4	Exclude 2009 and no trend	0.9600	0.0571	−2008.451	35.3%	12750
	5	Exclude 2009 and two trend terms	0.7965	0.0590	−1993.141	29.2%	12787
	6	Include rotavirus	1.0140	0.0534	−2319.427	37.6%	12647
	7	Include GI admissions	0.9100	0.0518	−2313.869	33.8%	12645

*Model descriptions: 1, includes term for RSV-positive identifications only; 2, includes a linear trend term; 3, includes quadratic trend term; 4, excludes 2009 data; 5, excludes 2009 data and includes 2 separate linear trend terms for before and after 2009; 6, includes rotavirus-positive identifications.
†Coefficient from negative binomial regression.
‡Bayes information criterion.
§Percentage of bronchiolitis and pneumonia admissions attributable to RSV.
¶Estimated RSV-associated admissions for bronchiolitis and pneumonia 2005–2013.

Appendix Table 5. KK Women's and Children's Hospital admissions for bronchiolitis and pneumonia by ward type*

Condition	Ward type	No. admissions (%)	Average LOS	Mean bill size, SGD	Daily bill size, SGD
Bronchiolitis	A	449 (28.4)	2.5	2,368	947
	B1	125 (7.9)	2.3	1,477	642
	B2+	244 (15.5)	2.3	925	402
	B2	98 (6.2)	2.2	554	252
	C	663 (42.0)	2.5	402	161
	Total	1,579 (100.0)			
Pneumonia (no complications)	A	378 (37.2)	2.9	2,853	984
	B1	118 (11.6)	2.6	1,696	652
	B2+	207 (20.4)	2.6	1,087	418
	B2	115 (11.3)	2.8	892	319
	C	197 (19.4)	3	544	181
	Total	1,015 (100.0)			
Pneumonia with complications	A	87 (38.3)	3.5	3,481	995
	B1	0 (0.0)	0	NA	NA
	B2+	45 (19.8)	3.5	1,295	370
	B2	0 (0.0)	0	NA	NA
	C	95 (41.9)	5.1	1,063	208
	Total	227 (100.0)			

*KKH admissions for bronchiolitis and pneumonia, July 1, 2016–June 30, 2017, billing by ward type. LOS, length of stay; NA, not applicable.

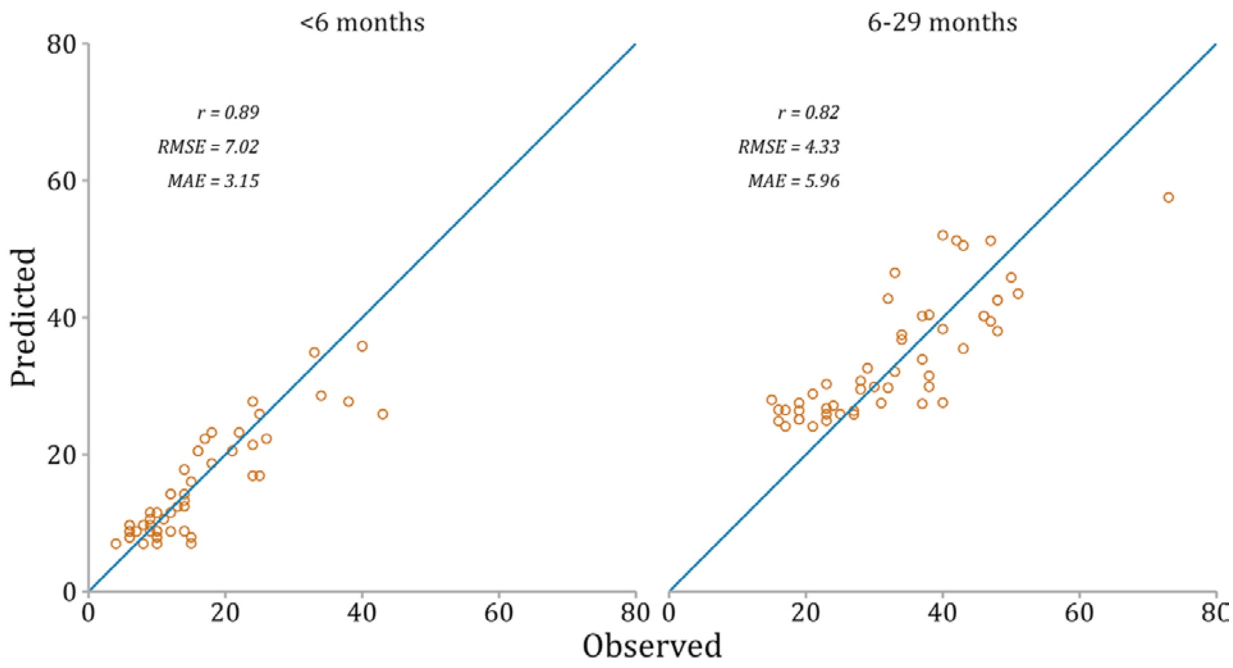

Appendix Figure 1. Correlation between observed and model-predicted weekly bronchiolitis and pneumonia admissions at KK Women's and Children's Hospital in 2014, by age group. R, correlation coefficient; RMSE, root mean squared error; MAE, mean absolute error.

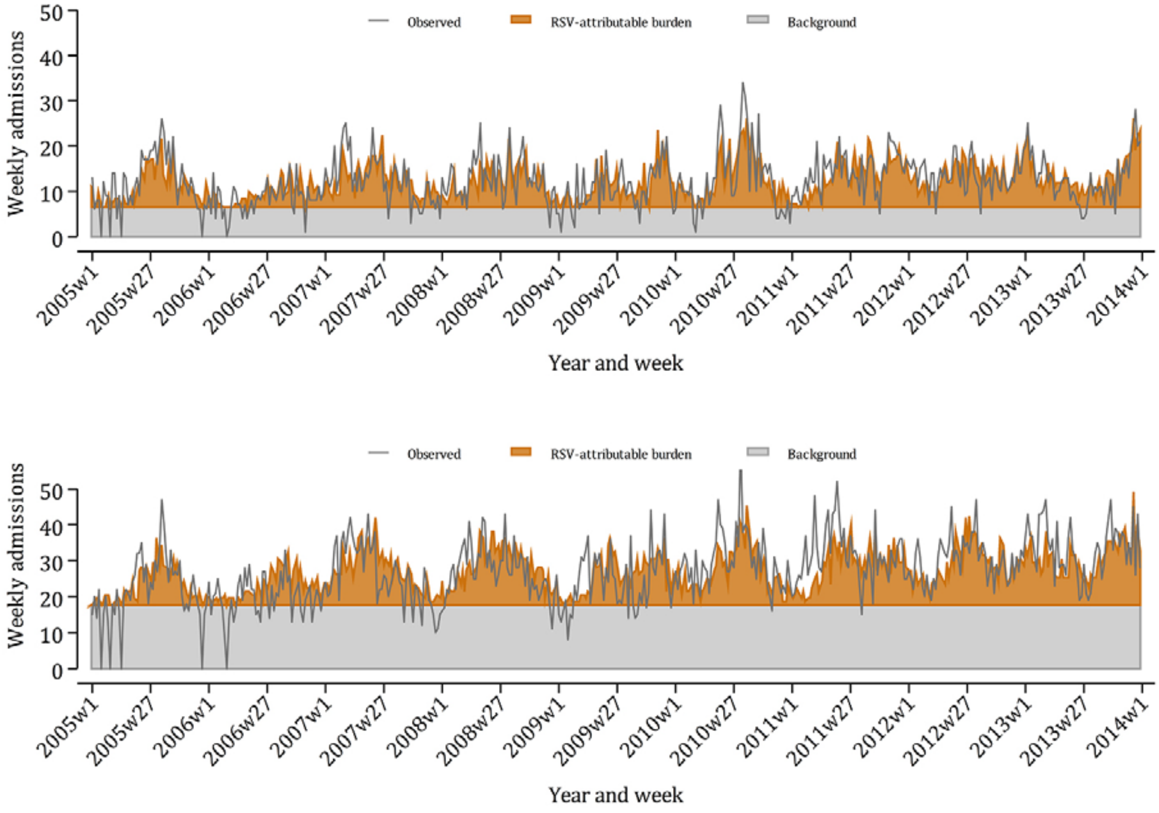

Appendix Figure 2. Observed and model-predicted weekly hospitalizations for bronchiolitis and pneumonia, KKH Women's and Children's Hospital, Singapore, 2005–2013. A) Children <6 months of age. B) Children 6–29 months of age. Gray line represents observed weekly bronchiolitis and pneumonia hospitalizations; orange area represents model-estimated RSV-attributable hospitalizations; gray area represents admissions attributable to other pathogens.

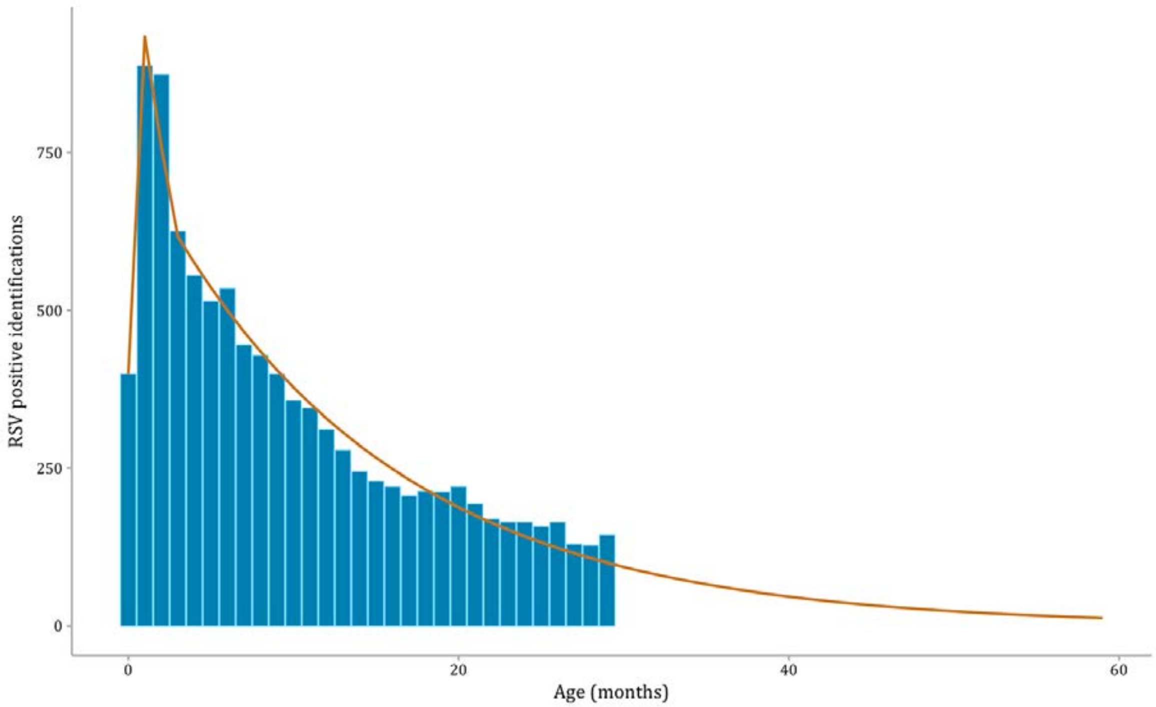

Appendix Figure 3. Age distribution of RSV-positive identifications at KK Women's and Children's Hospital. Blue bars indicate observed data; orange line indicates predicted values up to the age of 59 months from a logarithmic model.

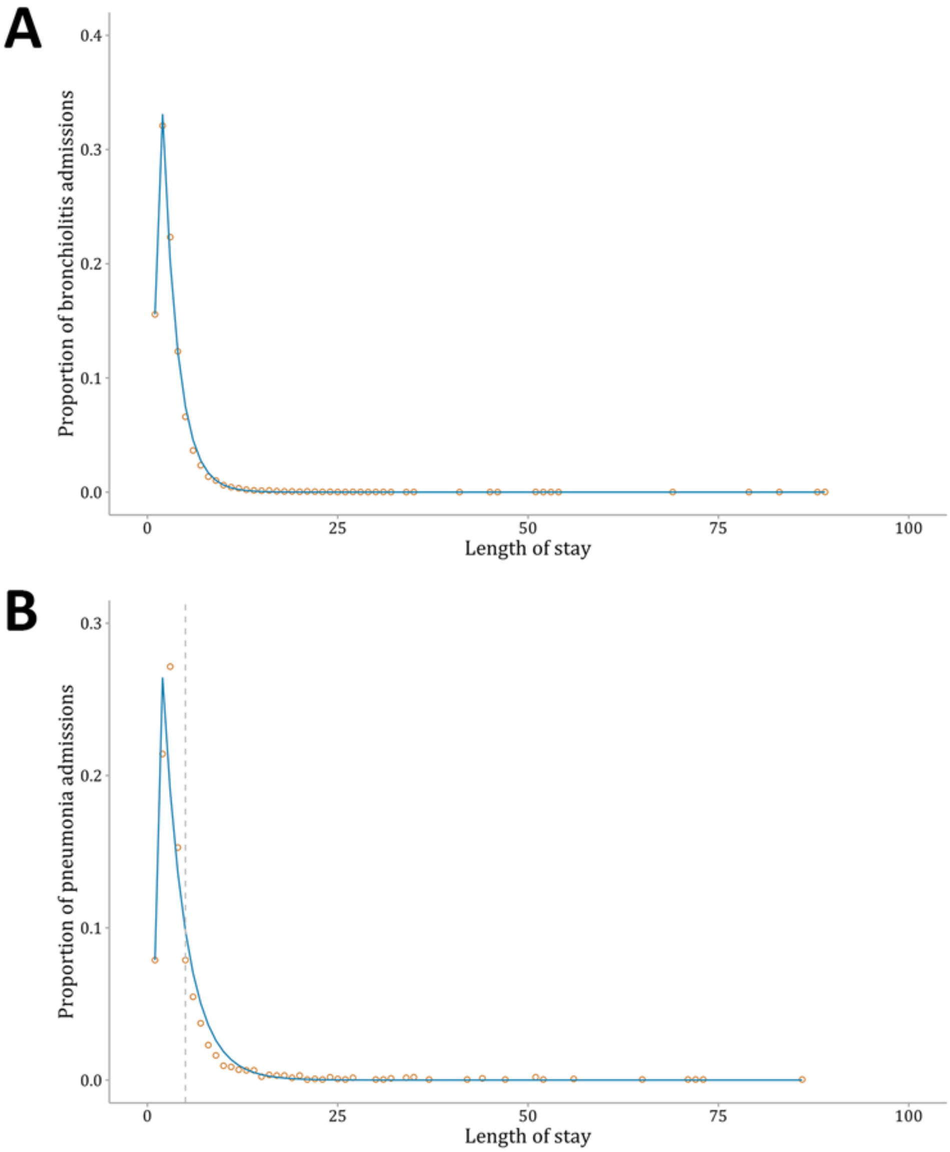

Appendix Figure 4. Distribution of length of stay at KK Women's and Children's Hospital for bronchiolitis (A) and pneumonia (B) admissions among children <30 months of age. Orange circles indicate observed data; blue lines indicate predicted values from a logarithmic model; dashed line (panel B) indicates cutoff of 5 days used to define pneumonia with complications. A total of 5 observations with length of stay >100 days were omitted from panel A.

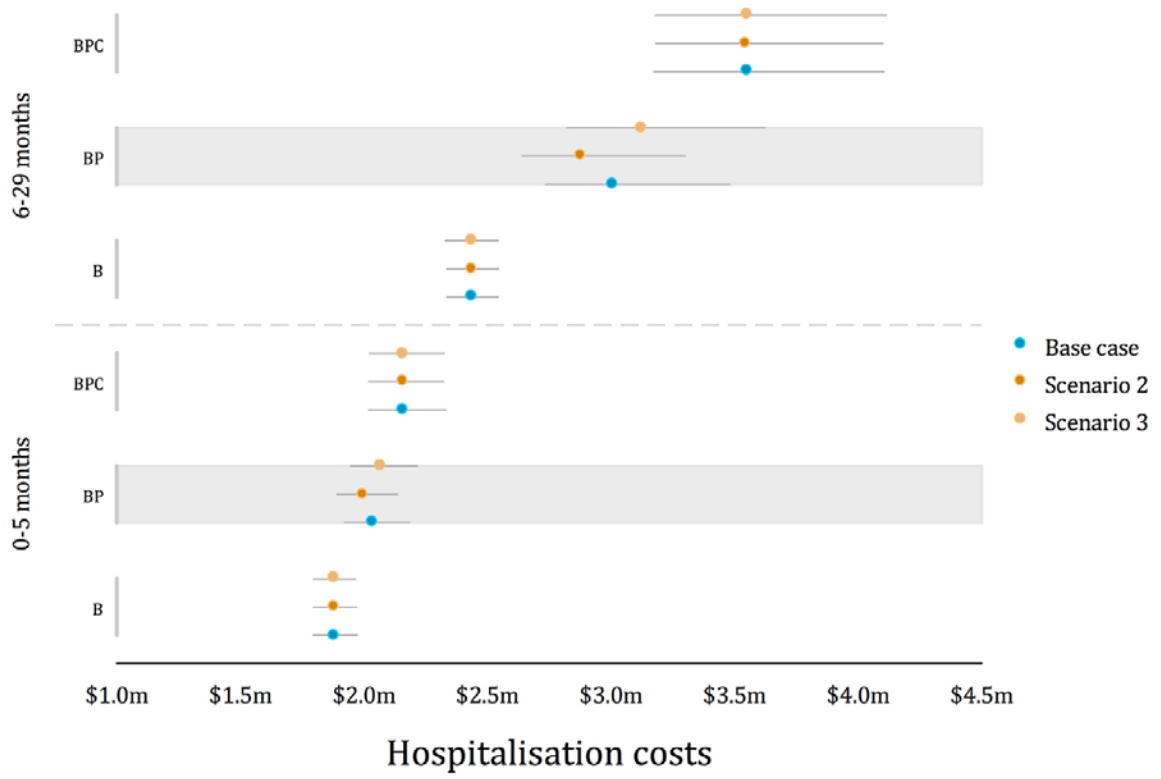

Appendix Figure 5. Impact of varying the definition of pneumonia with complications on estimated hospitalization costs. Pneumonia with complications is defined for 3 scenarios: base case, length of stay (LOS) >5 days; Scenario 2, LOS >4 days; Scenario 3, LOS >6 days.

Varying the definition of pneumonia with complications has little effect on the overall cost estimates because the daily hospitalization bill size is similar for pneumonia admissions with and without complications; the higher overall unit cost of admissions for pneumonia with complications is largely driven by longer hospital stays. B, bronchiolitis only; BP: bronchiolitis and pneumonia without complications, BPC, bronchiolitis, uncomplicated pneumonia, and pneumonia with complications.
